# Diversity of marine ostracods (Crustacea) from the inner Gulf of Thailand

**DOI:** 10.7717/peerj.21817

**Published:** 2026-10-26

**Authors:** Anisong Chitnarin, Lalita Weerachai, Marie-Béatrice Forel, Chatchalerm Ketwetsuriya

**Affiliations:** 1 School of Geotechnology, Institute of Engineering, Suranaree University of Technology, Mueang, Nakhon Ratchasima, Thailand; 2 School of Biological Sciences, Area of Ecology and Biodiversity, Swire Institute of Marine Science, Institute for Climate and Carbon Neutrality, and Musketeers Foundation, Institute of Data Science, University of Hong Kong, Hong Kong, China; 3 Centre de Recherche en Paléontologie - Paris, Museum National d’Histoire Naturelle, Paris, France; 4 Department of Earth Sciences, Faculty of Science, Kasetsart University, Bang Kane, Bangkok, Thailand

**Keywords:** Ostracoda, Crustaceans, Microcrustaceans, Seed shrimp

## Abstract

This study examined ostracod assemblages from surface sediments collected at four major river mouths—Mae Klong, Bang Pakong, Chao Phraya, and Tha Chin—and adjacent offshore sites in the Gulf of Thailand. Forty species are identified, including one new genus,* Aothai Forel, Chitnarin, Weerachai & Ketwetsuriya* gen. nov., with the type species *Aothai kresnae* sp. nov. Ostracod distribution shows a clear spatial differentiation between river-mouth and offshore environments. River-mouth assemblages are dominated by *Neocyprideis agilis* (Wouters, 2005) and are characterized by low salinity (less than 30 PSU), shallow depths, and fine-grained sediments. In contrast, offshore assemblages exhibit higher species diversity and are dominated by taxa such as *Keijella multisulcus* Whatley & Zhao, 1988, *Sinocytheridea impressa* (Brady, 1869), *Hemicytheridea cancellata* (Brady, 1868), *Stigmatocythere indica* (Jain, 1977), and *Neomonoceratina rhomboidea* (Brady, 1868). This study provides the first comprehensive documentation of modern ostracod assemblages from the inner Gulf of Thailand and establishes a baseline for future taxonomic, ecological, and palaeoenvironmental investigations in this region.

## Introduction

The Gulf of Thailand, located in Southeast Asia, is an epicontinental sea connected to the southern part of the South China Sea (Fig. 1A). The Gulf is a semi-enclosed basin, becoming shallower toward the north and deeper toward the southeast, with water depths ranging from about 10 to 80 m (*e.g.*, Sinsakul, 1992; Sojisuporn, Morimoto & Yanagi, 2010; Phattananuruch & Pokavanich, 2024). The northern coast, where Bangkok is located, is bordered by both industrial areas and mangrove ecosystems. Four major rivers—the Chao Phraya, Tha Chin, Mae Klong, and Bang Pakong—deliver large amounts of sediment into the Gulf.

**Figure 1:**
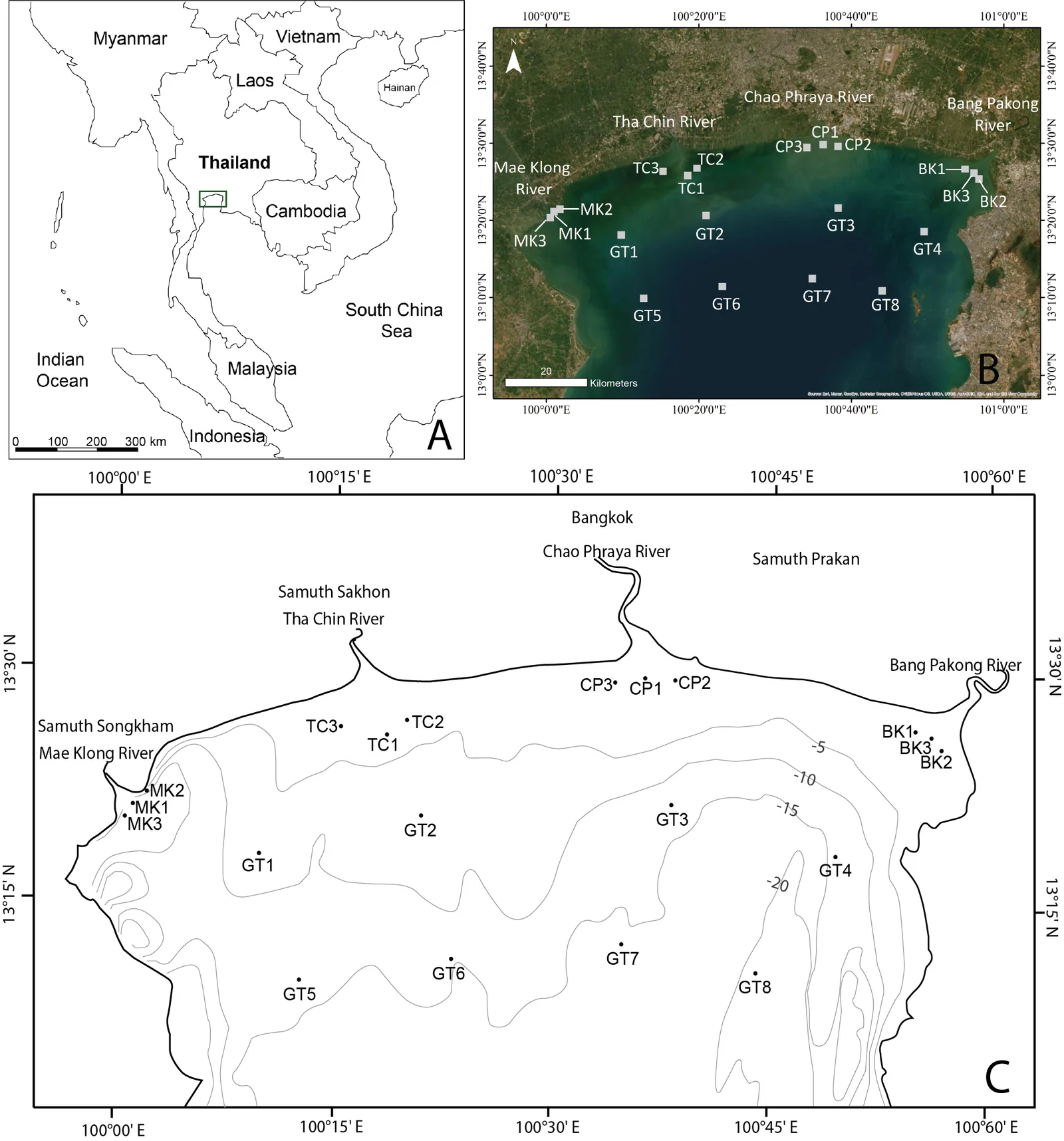
Locality maps. (A) Index map showing the position of the Gulf of Thailand. (B) Sampling localities (Map data © 2025 Google). (C) Localities of river-mouth samples (MK, Mae Khlong River; TC, Tha Chin River; CP, Chao Phraya River; BK, Bang Pakong River) and offshore marine stations (GT).

This northern part of the basin is bounded by the western and eastern coasts, forming a concave embayment known as the inner Gulf of Thailand (Fig. 1B). The inner Gulf is characterized by shallow bathymetry and gentle slopes from the coastline toward the central part of the basin, with depths generally ranging from 0 to 30 m (Fig. 1C). Among the river mouths, the Mae Klong estuary in the northwest is the shallowest area (0–10 m), whereas the Chao Phraya estuary in the central part is relatively deeper, reaching about 15–20 m (Yottiam et al., 2025).

Previous studies have shown that circulation patterns in the Gulf of Thailand vary seasonally and are controlled by monsoon winds (*e.g.*, Sojisuporn, Morimoto & Yanagi, 2010; Saramul, 2017; Phattananuruch & Pokavanich, 2024). As shown in Fig. 2, during the northeast monsoon (November to February), surface currents generally flow southwestward, whereas during the southwest monsoon (May to September), they reverse and flow toward the northeast (Sojisuporn, Morimoto & Yanagi, 2010). However, the concave shape of the inner Gulf tends to restrict the direct influence of open-ocean circulation from the outer Gulf. Sediment transportation and deposition in the inner Gulf are influenced by several factors, including river discharge, tides, monsoon winds, and oceanic currents. Surface-water salinity in the inner Gulf (approximately 29–30 ppt) is lower than that of the outer Gulf and the South China Sea (Sojisuporn, Morimoto & Yanagi, 2010).

**Figure 2:**
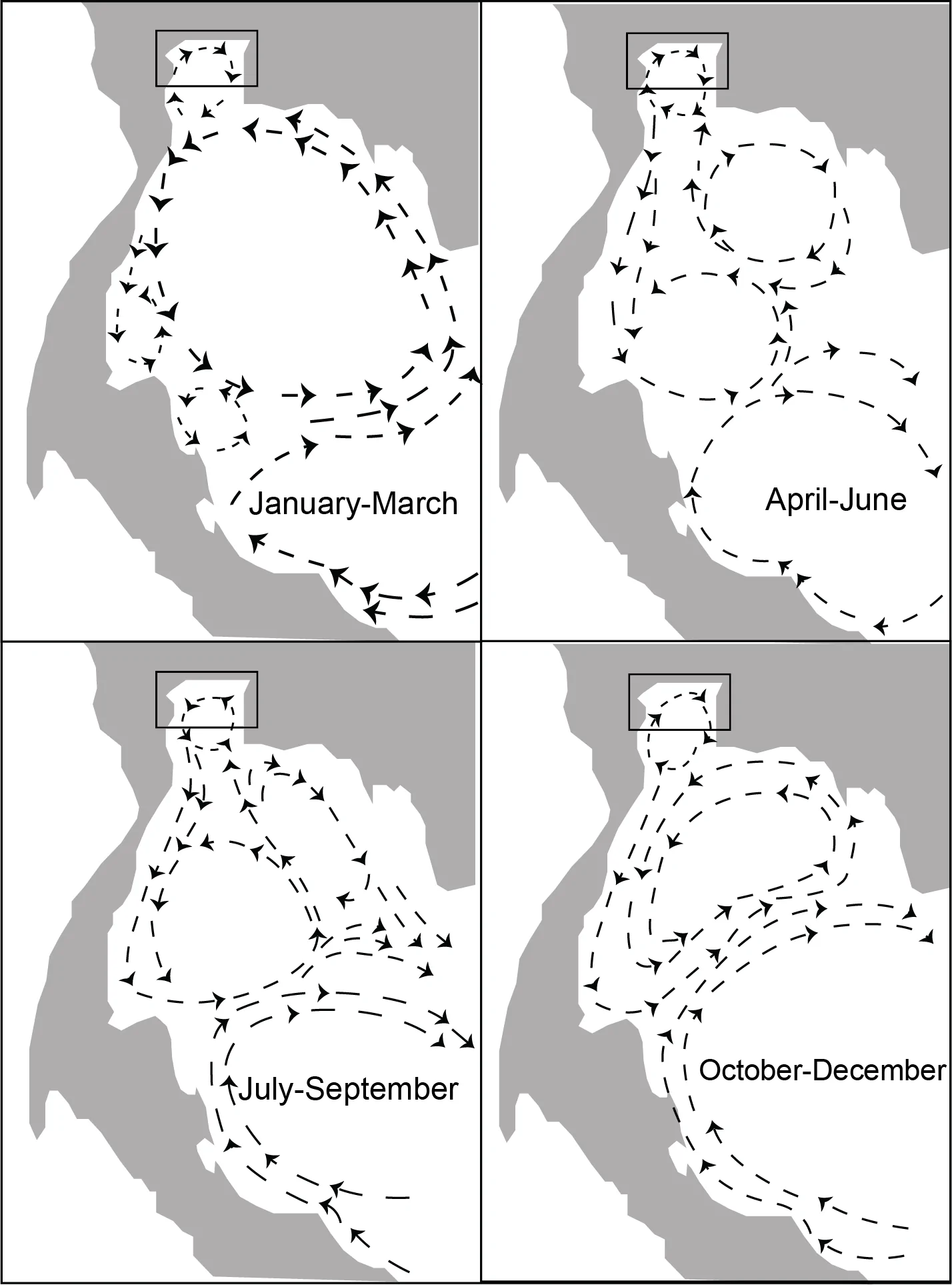
Seasonal circulation in the Gulf of Thailand (redrawn after Sojisuporn, Morimoto & Yanagi, 2010). The rectangle indicates the study area.

The inner Gulf of Thailand is important to the country’s economy due to its suitability for transportation and industrial development. In recent years, climate change and pollution have become increasing concerns in environmental planning and management (*e.g.*, Worakhunpiset, 2018; Phattananuruch & Pokavanich, 2024; Yottiam et al., 2025). Although the physical and hydrodynamic characteristics of the Gulf have been relatively well studied, the application of biological indicators—such as foraminifers, diatoms, and ostracods—for environmental assessment (*e.g.*, Frenzel & Boomer, 2005; Yasuhara et al., 2012; Benito, 2020; Parameswari et al., 2020; Huang et al., 2025; Huang et al., 2026) is still limited in Thailand.

Ostracods (small crustaceans) are good ecological indicators and are frequently used to monitor environmental changes and contamination (*e.g.*, Smith & Horne, 2002; Frenzel & Boomer, 2005; Hong et al., 2019; Tan et al., 2021; Schmitz et al., 2024; Schmitz et al., 2025). Although marine ostracods are known from the Indo-Pacific region (*e.g.*, Whatley & Zhao, 1987; Whatley & Zhao, 1988a; Whatley & Zhao, 1988b; Zhao & Whatley, 1989; Mostafawi, Colin & Babinot, 2005; Yamada, 2007; Niiyama et al., 2019; Tan et al., 2021), knowledge of their biodiversity and distribution in the Gulf of Thailand is limited. An investigation of shallow-water ostracod fauna near the mouth of the Mae Khlong River (northwestern part of the Gulf of Thailand) identified 34 species and reported that they are influenced by environmental factors such as salinity, dissolved oxygen, pH, and freshwater influx (Montenegro, Pugliese & Sciuto, 2004). Pugliese et al. (2006) later investigated the environmental conditions that influence ostracod distribution in the same area, demonstrating that the assemblages can serve as effective markers of river influence and human activities. Since then, no additional studies on ostracods have been reported from the Gulf of Thailand. In contrast, only two studies have been conducted along the Andaman coast of Thailand (Yamada, Terakura & Tsukawaki, 2014; Forel, 2021). The objective of this study is to document the diversity and occurrence of ostracods in the inner Gulf of Thailand, generating a baseline dataset to support future environmental monitoring and ecological applications.

## Materials and Methods

### Field investigation and sampling design

Sediment samples were collected from the inner Gulf of Thailand (GT), a shallow semi-enclosed marine basin influenced by major river discharges (Fig. 1B). Sampling stations were distributed across two environmental settings: river mouth (estuarine) stations and offshore marine stations (Figs. 1B and 1C). The geographic coordinates, water depth, and sediment characteristics of all stations are summarized in Table 1. Stations were classified as river mouth (estuarine) stations when located within the river–sea mixing zone and directly influenced by freshwater discharge, whereas offshore marine stations were located farther from the coastline and outside the immediate estuarine plume. This classification follows previous environmental studies of the Gulf of Thailand that distinguish estuarine and open-marine conditions based on hydrological influence and distance from river discharge (*e.g.*, Vibhatabandhu & Srithongouthai, 2022; Yottiam et al., 2025).

**Table 1:** Geographic coordinates, water depth, and sediment characteristics of sampling stations.

**Sample number**	**Latitude**	**Longitude**	**Depth (m)**	**Lithology**
GT1	13.301342 N	100.163838 E	−7	fine-grained sand, silt, clay
GT2	13.342762 N	100.349072 E	−7	fine-grained sand, silt, clay
GT3	13.358644 N	100.637518 E	−13	fine-grained sand, silt, clay
GT4	13.308565 N	100.826188 E	−14	fine-grained sand, silt, clay
GT5	13.164572 N	100.212529 E	−14	silt, clay
GT6	13.190306 N	100.384860 E	−15	fine-grained sand, silt, clay
GT7	13.208179 N	100.581236 E	−16	silt, clay
GT8	13.180637 N	100.734828 E	−23	silt, clay
MK1	13.351390 N	100.017223 E	−3	fine-grained sand, silt, clay
MK2	13.356393 N	100.029996 E	0	silt, clay
MK3	13.338333 N	100.008609 E	0	silt, clay
TC1	13.429445 N	100.308614 E	−2	silt, clay
TC2	13.445555 N	100.329169 E	−2	silt, clay
TC3	13.439164 N	100.255273 E	−3	silt, clay
CP1	13.496946 N	100.604723 E	−2	clay
CP2	13.493059 N	100.636943 E	−1	clay
CP3	13.491113 N	100.569446 E	−1	clay
BK1	13.434994 N	100.934997 E	−8	clay
BK2	13.422501 N	100.945837 E	−8	clay
BK3	13.443611 N	100.915555 E	−2	clay

**Notes.**

GT offshore samples within the inner Gulf of Thailand MK Mae Khlong River Mouth TC Tha Chin River Mouth CP Chao Phraya River Mouth BK Bang Pakong River Mouth

The four major river systems that discharge into the inner Gulf of Thailand were investigated: Mae Khlong (MK), Tha Chin (TC), Chao Phraya (CP), and Bang Pakong (BK), ordered from west to east. These rivers represent the major freshwater inputs into the northern Gulf and were selected to examine ostracod assemblages under varying estuarine influences. In contrast, offshore marine stations (GT) were selected to represent varying geographic locations and water depths across the inner Gulf of Thailand.

Sediment collection was conducted during two distinct periods in 2023 to evaluate potential seasonal variations: May (representing the summer/dry season transition) and December (representing the winter/NE monsoon season). These two sampling periods were intended to provide a preliminary comparison between contrasting seasonal conditions. However, because sampling was conducted in a single year, the results should be interpreted as temporal snapshots rather than a full characterization of seasonal variability.

At each of the four river mouths, three nearby sampling locations were established along the estuarine zone to capture small-scale spatial variability. At each location, three separate grab samples were collected to assess within-site variability. These are labeled sequentially (*e.g.*, MK1, MK2, and MK3). In addition to the estuarine stations, eight offshore marine stations (GT1–GT8) were established across the inner Gulf of Thailand to represent typical marine conditions away from direct river influence. These offshore stations were selected to cover a range of water depths and geographic positions within the study area. At each marine station, surface sediments and deeper sediment layers (10 cm depth) were collected using a grab sampler.

### Sediment collection

Sediments were collected using a Van Veen grab sampler deployed from a research vessel. Each grab sample retrieved approximately 7–10 kg of unconsolidated sediment from the seabed. For offshore stations (GT1–GT8), two sediment subsamples were taken from each grab: surface sediment (0–2 cm), representing the most recently deposited material, and subsurface sediment (∼10 cm), representing slightly older layers. This distinction was made to evaluate potential differences between recently deposited and slightly deeper sediment horizons.

At the river mouth stations, each grab was treated as an independent replicate, yielding three replicate samples at each location. Samples were labeled sequentially according to river system, location, and replicate number (*e.g.*, MK1-1, MK1-2, MK1-3). In this coding system, the first letter code indicates the river system. The number following the code indicates the sampling location within that river system. The final number represents the replicate sample from that location. In total, 16 samples were obtained from the offshore stations (eight stations × two sediment horizons), and 72 samples were collected from the river mouth environments (four rivers × three locations × three replicates × two sampling periods). Sediment samples were stored in labeled plastic bags and transported to the laboratory for further processing. At each station, the physical and chemical properties of seawater, including temperature (^∘^C), dissolved oxygen (mg/L), pH, and salinity (PSU), were measured at both the surface and the deep levels to characterize the benthic habitat. The environmental variables of seawater at the sampling stations are shown in Tables 2 and 3 and Fig. 3.

### Sample processing method

All sediment samples were soaked with water, washed through a 0.063 mm (63 µm) sieve, and dried. Ostracod shells were picked under an optical binocular microscope, and both carapaces and isolated valves were counted. A single individual was defined as one entire carapace, one pair of single left and right valves, and one individual valve with no matching counterpart. The specimens were then pictured using a JEOL/JSM-6010LV scanning electron microscope at the Suranaree University of Technology (Nakhon Ratchasima, Thailand) for detailed identification. Ostracods were recovered from the offshore samples; however, only 34 samples out of 72 from the river mouths yielded ostracods (Tables S1–S2). The barren samples (May) include MK3-1, TC1-1, TC1-2, TC1-3, TC2-1, TC2-3, TC3-1, TC3-3, CP1-1, CP1-2, CP1-3, CP2-1, CP2-2, CP2-3, CP3-1, BK2-3 and BK3-3. The barren samples (December) include MK1-1, MK1-2, MK2-1, MK2-2, MK3-1, MK3-2, TC1-1, TC1-2, TC1-3, TC2-1, TC2-2, TC3-1, TC3-2, CP1-1, CP1-2, CP2-1, CP2-2, CP2-3, CP3-1, CP3-2, CP3-3, BK1-1, BK1-2, BK2-1, BK2-2, BK3-1 and BK3-2. In the present work, only carapaces and valves were recovered, and no specimens with appendages or soft parts were found. Identifications, therefore, rely entirely on carapace and valve characters.

The ostracod classification follows the general scheme of Moore (1961), as well as updated classifications such as those of Hartmann & Puri (1974), Martens & Horne (2009), and the World Ostracoda Database (Brandão et al., 2026). Forty species belonging to twenty nine genera are identified (Table 4, Figs. 4–9), including a new genus (Figs. 6–8). Most species found in this study were already known from the Indo-Pacific region, and in the following, taxonomy is limited to the description of a new genus, *Aothai* gen. nov., and the most abundant species *Neocyprideis agilis* (Wouters, 2005).

**Table 2:** Environmental parameters of seawater at river mouth sampling stations (May and December 2023).

**Environmental parameters**	**MK1**	**MK2**	**MK3**	**TC1**	**TC2**	**TC3**	**CP1**	**CP2**	**CP3**	**BK1**	**BK2**	**BK3**
May 2023												
Temperature (°C)	31.7	29.7	32.3	30.8	30.8	31.8	33	32	34.2	33.9	35.1	33.3
Dissolved oxygen (mg/L)	5.6	6.1	5.8	6.0	5.2	5.7	7.3	7.3	7.2	4.8	4.7	4.4
pH	7.69	7.83	7.73	7.94	7.81	7.79	8.07	7.96	10.73	7.38	7.9	7.57
Salinity (PSU)	20.1	23.48	9.18	25.28	25.31	24.29	19.5	9.56	21.87	25.58	25.7	25.39
December 2023												
Temperature (°C)	27.2	28	27.8	29.9	29.2	30.3	28.3	28.7	28.5	27.3	27.2	27.6
Dissolved oxygen (mg/L)	5.8	6.8	7.3	7.3	6.0	5.0	6.0	6.0	7.1	4.9	5.4	5.4
pH	7.77	8.05	7.74	8.57	8.72	8.64	8.09	8.56	8.27	7.55	7.72	7.77
Salinity (PSU)	25.63	28.32	27.17	29.24	28.87	23.25	30.12	30.92	30.58	20.58	23.96	21.29

**Table 3:** Environmental parameters of seawater at the inner Gulf of Thailand sampling stations (May and December 2023).

**Environmental parameters**	**1 meter deep from the water surface**								**1 meter above the sea floor**							
	GT1	GT2	GT3	GT4	GT5	GT6	GT7	GT8	GT1	GT2	GT3	GT4	GT5	GT6	GT7	GT8
May 2023																
Depth (m)	1.0	1.0	1.0	1.0	1.0	1.0	1.0	1.0	9.5	8.5	14.0	15.0	17.6	15.7	16.7	22.0
Temperature (°C)	31.2	31.5	30.1	32.0	32.2	32.6	32.2	31.8	30.5	31.5	30.6	31.9	31.2	31.6	31.7	31.8
Dissolved oxygen (mg/L)	5.57	6.30	5.98	5.63	5.53	5.81	6.08	5.75	5.59	6.28	4.16	5.46	5.26	5.41	5.01	4.87
pH	8.48	8.6	8.53	8.61	8.42	8.45	8.46	8.45	8.52	8.61	8.47	8.56	8.51	8.41	8.47	8.40
Salinity (PSU)	31.1	30.5	30.1	30.5	32.1	31.6	30.6	31.4	31.1	30.5	30.6	30.6	31.8	31.6	31.4	31.5
December 2023																
Depth (m)	1.0	1.0	1.0	1.0	1.0	1.0	1.0	1.0	10.0	10.0	15.0	16.0	18.0	17.5	19.0	24.0
Temperature (°C)	33.3	30.1	31.0	30.7	33.0	32.6	31.8	32.1	31.7	29.8	30.9	30.5	32.0	31.7	31.2	32.0
Dissolved oxygen (mg/L)	8.66	8.28	7.35	7.11	7.24	7.1	7.04	7.09	4.67	7.2	7.04	6.94	6.68	6.91	6.91	6.88
pH	7.79	8.05	8.28	8.18	8.05	8.24	8.24	8.34	7.89	8.14	8.27	8.18	8.19	8.28	8.27	8.13
Salinity (PSU)	28.2	29.4	33.2	32.7	32.4	32.8	31.8	31.9	31.7	31.8	33.4	33.0	32.5	33.0	31.2	32.0

**Figure 3:**
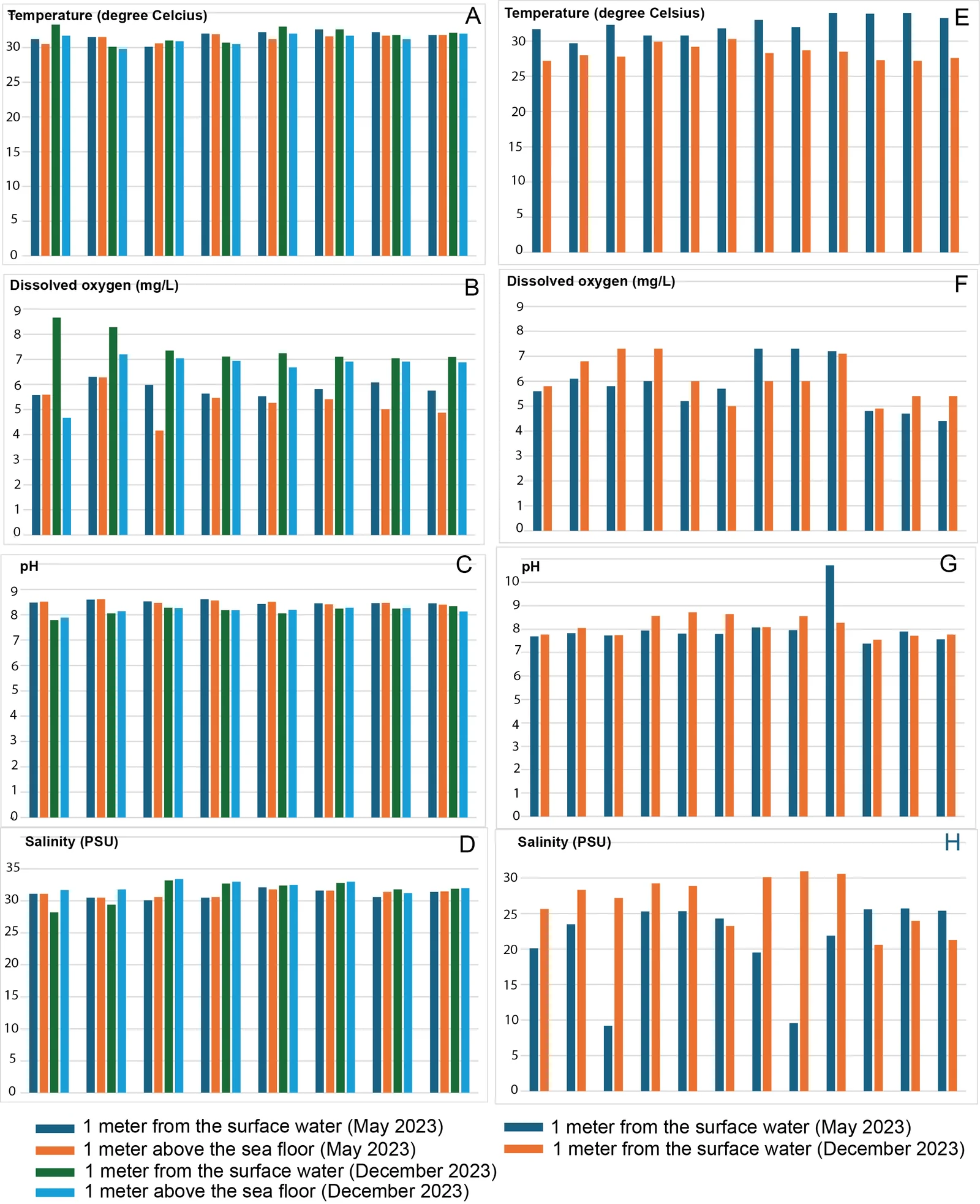
Environmental parameters of the sampling localities. (A–D) Localities in the Gulf of Thailand (GT1-GT8). (E–H) River mouth localities (MK, Mae Khlong River; TC, Tha Chin River; CP, Chao Phraya River; BK, Bang Pakong River).

**Table 4:** List of ostracods from the river-mouth and the inner Gulf of Thailand sampling stations.

Ostracods	Figures	Sample numbers
Subclass PODOCOPA Müller, 1894		
Order PODOCOPIDA Müller 1894		
Suborder PLATYCOPINA Sars, 1866		
Superfamily CYTHERELLOIDEA Sars 1866		
Family CYTHERELLIDAE Sars 1866		
Genus *Cytherella Jones, 1849*		
*Cytherella* sp.	Figures 4A–4B	GT1, GT2, GT3, GT4, GT5, GT6, GT7, GT8, MK2, CP3, BK1, BK2
*Cytherella incohata Zhao & Whatley, 1989*	Figure 4C	GT1, GT2, GT3, GT4, GT5, GT6, GT7, GT8
Suborder PODOCOPINA Sars, 1866		
Superfamily BAIRDIOIDEA Sars, 1888		
Family BAIRDIIDAE Sars, 1888		
Subfamily BAIRDIINAE Sars, 1888		
Genus *Paranesidea Maddocks, 1969*		
*Paranesidea* sp.	Figures 4D–4F	GT5
Superfamily CYTHEROIDEA Baird, 1850		
Family CYTHERIDEIDAE Baird, 1850		
Genus *Sinocytheridea Hou, 1982*		
*Sinocytheridea impressa (Brady, 1869)*	Figure 4G	GT1, GT2, GT3, GT4, GT5, GT6, GT7, GT8
Genus *Javanella* Kingma, 1948		
*Javanella kendengensis* Kingma, 1948	Figure 4H	GT6, GT8
Subfamily CYTHERIDEINAE Sars, 1925		
Genus *Neocyprideis Apostolescu, 1956*		
*Neocyprideis agilis* (Wouters, 2005)	Figures 4I–4U	MK1, MK2, MK3, TC3, CP1, CP3, BK1, BK2, BK3
Family BYTHOCYTHERIDAE Sars, 1926		
Subfamily BYTHOCYTHERINAE Sars, 1926		
Genus *Bythoceratina Hornibrook, 1952*		
*Bythoceratina* sp.	Figure 5A	GT3, GT5, GT6, GT8, BK2
Family LEPTOCYTHERIDAE Hanai, 1957		
Genus *Tanella Kingma, 1948*		
*Tanella gracilisKingma, 1948*	Figure 5B	GT2, GT3, GT5, GT6, GT7, GT8, MK1, MK2, MK3, TC3
Family LOXOCONCHIDAE Sars, 1925		
Genus *Loxoconcha* Sars, 1866		
*Loxoconcha* sp.	Figure 5C	GT6, GT7, GT8
Genus *Aothai Forel, Chitnarin, Weerachai, Ketwetsuriya gen. nov.*		
*Aothai kresnae Forel, Chitnarin, Weerachai, Ketwetsuriya gen. nov. sp. nov.*	Figure 6	GT3, MK1, MK2, CP1, BK2
Family PARADOXOSTOMATIDAE Brady & Norman, 1889		
Genus *Paradoxostoma Fischer, 1855*		
*Paradoxostoma* sp.	Figures 5D–5E	GT1, GT3, GT6, GT7, GT8
Family CYTHERIDAE Baird, 1850		
Subfamily SCHIZOCYTHERINAE Mandelstam, 1959		
Genus *Neomonoceratina Kingma, 1948*		
*Neomonoceratina rhomboidea (Brady, 1868)*	Figure 5J	GT1, GT2, GT3, GT4, GT5, GT6, GT7, GT8, MK2
*Neomonoceratina mediterranea mediterranea (Ruggieri, 1953)*	Figure 5K	GT1, GT3, GT4, GT6, GT7, GT8, MK3, TC3, BK2, BK3
*Neomonoceratina mediterranea malayensis Zhao & Whatley, 1988*	Figure 5L	MK2, CP1, CP3, BK1, BK2, BK3
Family HEMICYTHERIDAE Puri, 1953		
Genus *Caudites Coryell & Fields, 1937*		
*Caudites javanus (Kingma, 1948)*	Figures 5F, 5I	GT7, GT8, MK1, MK2, MK3, CP3
Genus *Hemicytheridea Kingma, 1948*		
*Hemicytheridea reticulata Kingma, 1948*	Figure 9A	GT1, GT3, GT4, GT5, GT6, GT7, GT8, MK3
*Hemicytheridea cancellata (Brady, 1868)*	Figures 9B–9C	GT1, GT3, GT4, GT5, GT6, GT7, GT8, MK1, MK2, MK3, TC1, CP3
Family TRACHYLEBERIDIDAE Sylvester-Bradley, 1948		
Genus *Paracathaycythere Whatley & Zhao, 1991*		
*Paracathaycythere* cf. *costaereticulata*	Figures 5G–5H	MK1, MK2, BK2, BK3
Genus *Neocytheretta Morkhoven, 1963*		
*Neocytheretta murilineata Zhao & Whatley, 1989*	Figures 9D, 9E	GT3, GT4, GT6, GT7, GT8
*Neocytheretta* sp.	Figures 9G, 9H	GT4, MK1, MK2
Genus *Stigmatocythere Siddiqui, 1971*		
*Stigmatocythere bona Chen in Hou et al., 1982*	Figure 5M	GT1, GT2, GT3, GT4, GT5, GT6, GT7, GT8, MK1, MK2, TC3, CP3, BK1
*Stigmatocythere indica (Jain, 1977)*	Figures 5N–5O	GT1, GT2, GT3, GT4, GT5, GT6, GT7, GT8
*Stigmatocythere* sp.	Figure 9R	MK1
Genus *Atjehella Kingma, 1948*		
*Atjehella* sp.	Figures 9F, 9I	GT1, GT2, GT3, GT4, GT5, GT6, GT7, GT8
Genus *Keijella Ruggieri, 1967*		
*Keijella gonia Whatley & Zhao, 1989*	Figure 5P	GT1, GT2, GT3, GT4, GT5, GT6, GT7, GT8, MK1, MK2, MK3, TC3, BK1, BK2
*Keijella multisulcus Whatley & Zhao, 1988*	Figure 5Q	GT1, GT2, GT3, GT4, GT5, GT6, GT7, GT8, MK1, MK2, MK3, TC3, CP3, BK2
Genus *Lankacythere Bhatia & Kumar, 1979*		
*Lankacythere multiforaMostafawi, 1992*	Figure 9J	
*Lankacythere* sp.	Figures 9K, 9L	GT5
Genus *Pistocythereis Gou in Gou, Zheng & Huang, 1983*		GT4, GT6, GT8
*Pistocythereis subovata Gou in Gou, Zheng & Huang, 1983*	Figures 9M, 9N	GT3, GT5, GT7, GT8
*Pistocythereis bradyformis (Ishizaki, 1968) Gou in Gou, Zheng & Huang, 1983*		GT5
Genus *Venericythere Mostafawi, 1992*		
*Venericythere papuensis (Brady, 1880)*	Figure 9O	GT1, GT3, GT5, GT6, GT7
Genus *Hemikrithe Bold, 1950*		
*Hemikrithe orientalis Bold, 1950*	Figure 5R	GT1, GT4, GT5, GT6, GT7, MK1, CP3, BK2
Genus *Carinocythereis Ruggieri, 1956*		
*Carinocythereis* sp.	–	MK1
Family NEOCYTHERIDEIDIDAE *Puri, 1957*		
Genus *Copytus Skogsberg, 1939*		
*Copytus posterosulcus Wang, 1985*	Figures 9P, 9Q	GT3, GT5, GT6, GT7, GT8
Suborder CYPRIDOCOPINA Jones, 1901		
Superfamily CYPRIDOIDEA Baird, 1845		
Family CANDONIDAE Kaufmann, 1900		
Genus *Aglaiocypris Sylvester-Bradley, 1947*		
*Aglaiocypris pellucida Mostafawi, 2003*	Figure 5S	GT1, GT2, GT3, GT4, GT5, GT6, GT7, GT8, TC2, TC3, CP3
Superfamily PONTOCYPRIDOIDEA Müller, 1894		
Family PONTOCYPRIDIDAE Müller, 1894		
Genus *PropontocyprisSylvester-Bradley, 1947*		
*Propontocypris bengalensis Maddocks, 1969*	Figure 5T	GT1, GT2, GT3, GT4, GT5, GT6, GT7, GT8, MK1, CP3, BK1
Superfamily CYPRIDOIDEA Baird, 1845		
Family CANDONIDAE Kaufmann, 1900		
Genus *Phlyctenophora Brady, 1880*		
*Phlyctenophora orientalis (Brady, 1868)*	Figure 9S	GT1, GT2, GT4, GT5, GT6, GT7, GT8
Family PARACYPRIDIDAE Sars, 1923		
Subfamily PARACYPRIDINAE Sars, 1923		
Genus *Paracypris Sars, 1866*		
*Paracypris*? sp.	Figure 9T	GT5, GT7, GT8
Gen indet.	Figure 9U	CP3, BK1

**Figure 4:**
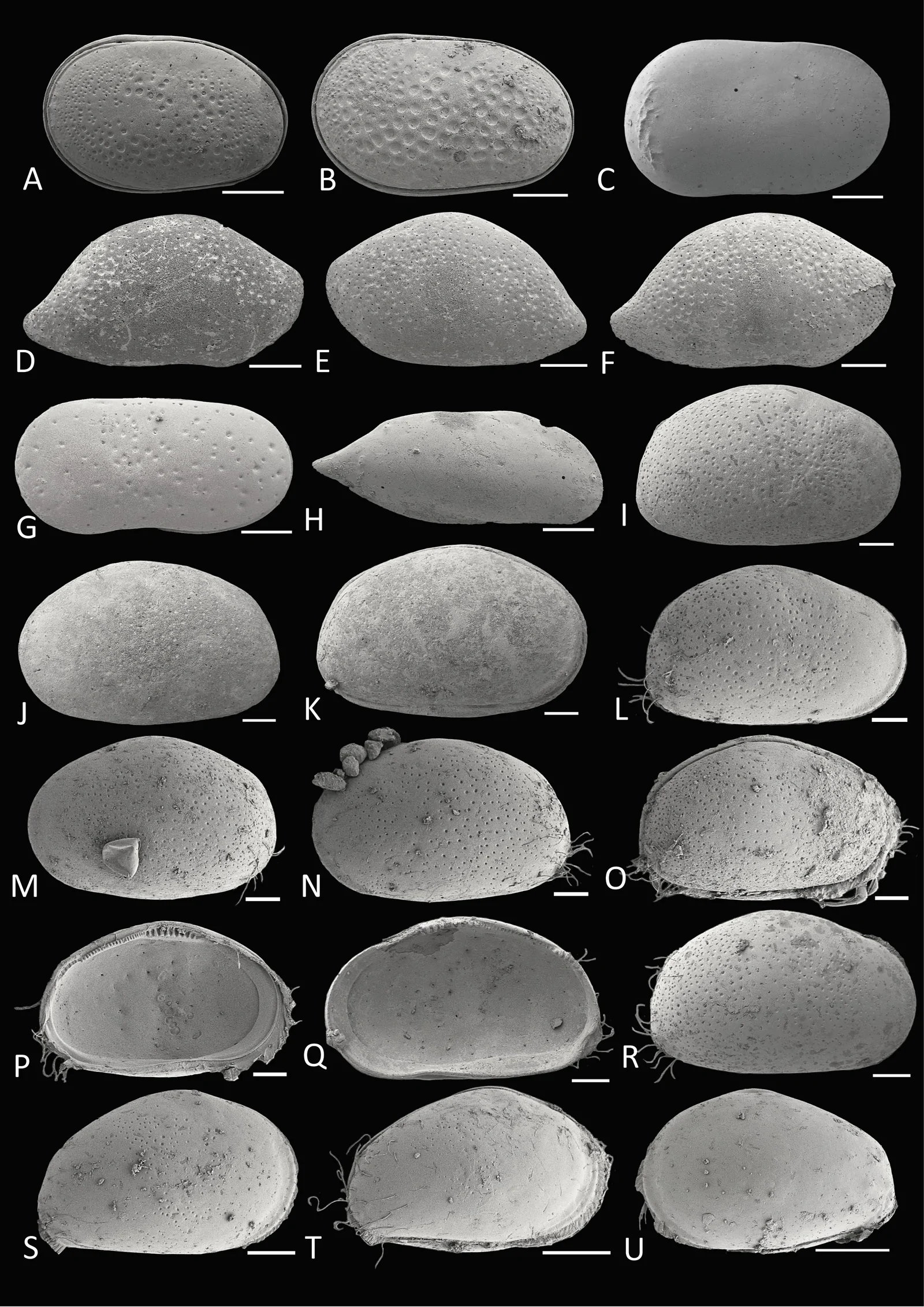
Scanning Electron Microscopic photographs of ostracods from the inner Gulf of Thailand. (A–B) *Cytherella* sp. (A) Right view of a carapace, sample GT1, SUT-25KU-001. (B) Right view of the carapace, sample GT2, SUT-25KU-140. (C) *Cytherella incohata* Zhao & Whatley, 1989, left valve, sample GT8, SUT-25KU-004. (D–F) *Paranesidea* sp. (D) Right valve, sample GT5, SUT-25KU-005. (E) Right valve, sample GT5, SUT-25KU-006. (F) Right valve, sample GT5, SUT-25KU-007. (G) *Sinocytheridea impressa* (Brady, 1869), left valve, sample GT1, SUT-25KU-011. (H) *Javanella kendengensis* Kingma, 1948, right valve, sample GT6, SUT-25KU-015. (I–U) *Neocyprideis agilis* (Wouters, 2005). (I) Right valve, sample MK1, SUT-25KU-016. (J) Left valve, sample MK1, SUT-25KU-017. (K) Right lateral view of the complete carapace, sample MK2, SUT-25KU-022. (L) Right valve, sample BK3, SUT-25KU-170. (M) Left valve, sample BK3, SUT-25KU-173. (N) Left valve, sample BK3, SUT-25KU-175. (O) Right lateral view of the complete carapace, sample BK3, SUT-25KU-163. (P) Internal view of the left valve, sample BK3, SUT-25KU-177. (Q) Internal view of the right valve, sample BK1, SUT-25KU-026. (R) Right valve, sample BK1, SUT-25KU-027. (S) Right valve, juvenile, sample BK3, SUT-25KU-157. (T) Right lateral view of the complete carapace, juvenile, sample BK3, SUT-25KU-152. (U) Left lateral view of the complete carapace, sample BK3, SUT-25KU-151. Scale bars = 0.1 mm.

The SEM photographs presented in Figs. 4–6 and 9 are selected from the species that may or may not have been previously recorded in Thailand but are illustrated here to make future references. All specimens and sediments are stored in the Micropaleontology Collections of Suranaree University of Technology, Nakhon Ratchasima, Thailand, under collection numbers SUT-25KU-001 to SUT-25KU-200.

The electronic version of this article in Portable Document Format (PDF) will represent a published work according to the International Commission on Zoological Nomenclature (ICZN), and hence the new names contained in the electronic version are effectively published under that Code from the electronic edition alone. This published work and the nomenclatural acts it contains have been registered in ZooBank, the online registration system for the ICZN. The ZooBank LSIDs (Life Science Identifiers) can be resolved and the associated information viewed through any standard web browser by appending the LSID to the prefix http://zoobank.org/. The LSID for this publication is: urn:lsid:zoobank.org:pub:083A1912-6302-4BCA-A412-D6EB0753FDDB. The online version of this work is archived and available from the following digital repositories: PeerJ, PubMed Central SCIE and CLOCKSS.

**Figure 5:**
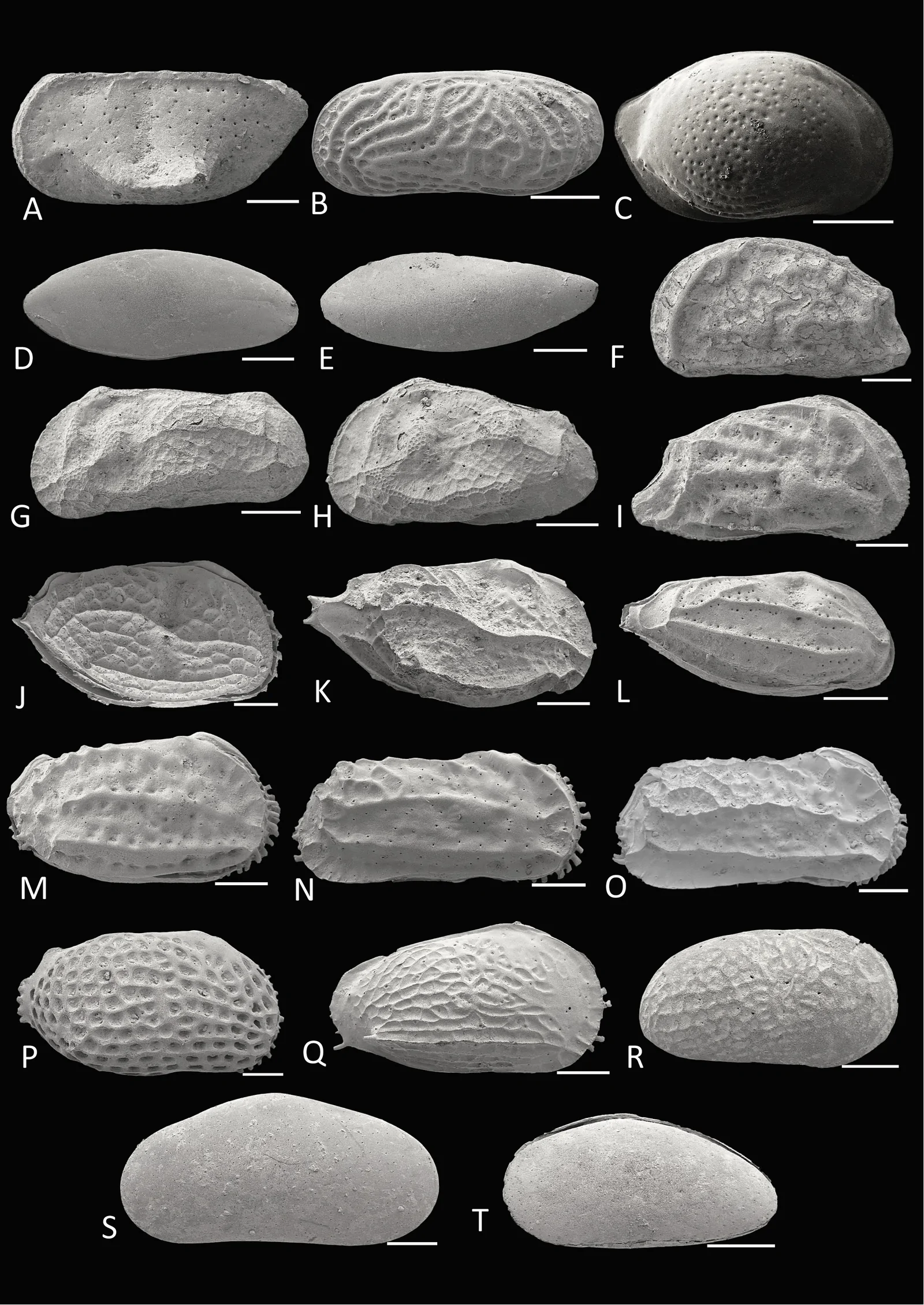
Scanning electron microscopic photographs of ostracods from the inner Gulf of Thailand. (A) *Bythoceratina* sp., left valve, sample GT5, SUT-25KU-030. (B) *Tanella gracilis* Kingma, 1948, right valve, sample GT8, SUT-25KU-032. (C) *Loxoconcha* sp., left valve, sample GT7, SUT-25KU-035. (D–E) *Paradoxostoma* sp. (D) Right valve, sample GT7, SUT-25KU-037. (E) Left lateral valve, sample GT1, SUT-25KU-038. (F, I) *Caudites javanus* (Kingma, 1948). (F) Left valve, sample MK1, SUT-25KU-052. (I) Right valve, sample MK1, SUT-25KU-054. (G–H) *Paracathaycythere* cf. *costaereticulata*. (G) Left valve, sample MK1, SUT-25KU-050. (H) Left valve, sample BK2, SUT-25KU-051. (J) *Neomonoceratina rhomboidea* (Brady, 1868), right lateral view of complete carapace, sample GT1, SUT-25KU-040. (K) *Neomonoceratina mediterranea mediterranea* (Ruggieri, 1953), right valve, sample GT4, SUT-25KU-045. (L) *Neomonoceratina mediterranea malayensis* Zhao & Whatley, 1988, right valve, sample CP3, SUT-25KU-049. (M) *Stigmatocythere bona* Chen in Hou et al., 1982, right lateral view of complete carapace, sample CP3, SUT-25KU-078. (N–O) *Stigmatocythere indica* (Jain, 1977). (N) right valve, sample GT6, SUT-25KU-074. (O) Right valve, sample GT1, SUT-25KU-079. (P) *Keijella gonia* Whatley & Zhao, 1989, right valve, sample GT1, SUT-25KU-083. (Q) *Keijella multisulcus* Whatley & Zhao, 1988 , right valve, sample GT1, SUT-25KU-089. (R) *Hemikrithe orientalis* Bold, 1950, left view of a carapace, sample GT1, SUT-25KU-098. (S) *Aglaiocypris pellucida* Mostafawi, 2003, right valve, sample GT2, SUT-25KU-103. (T) *Propontocypris bengalensis* Maddocks, 1969, right lateral view of complete carapace, sample GT1, SUT-25KU-106. Scale bars = 0.1 mm.

**Figure 6:**
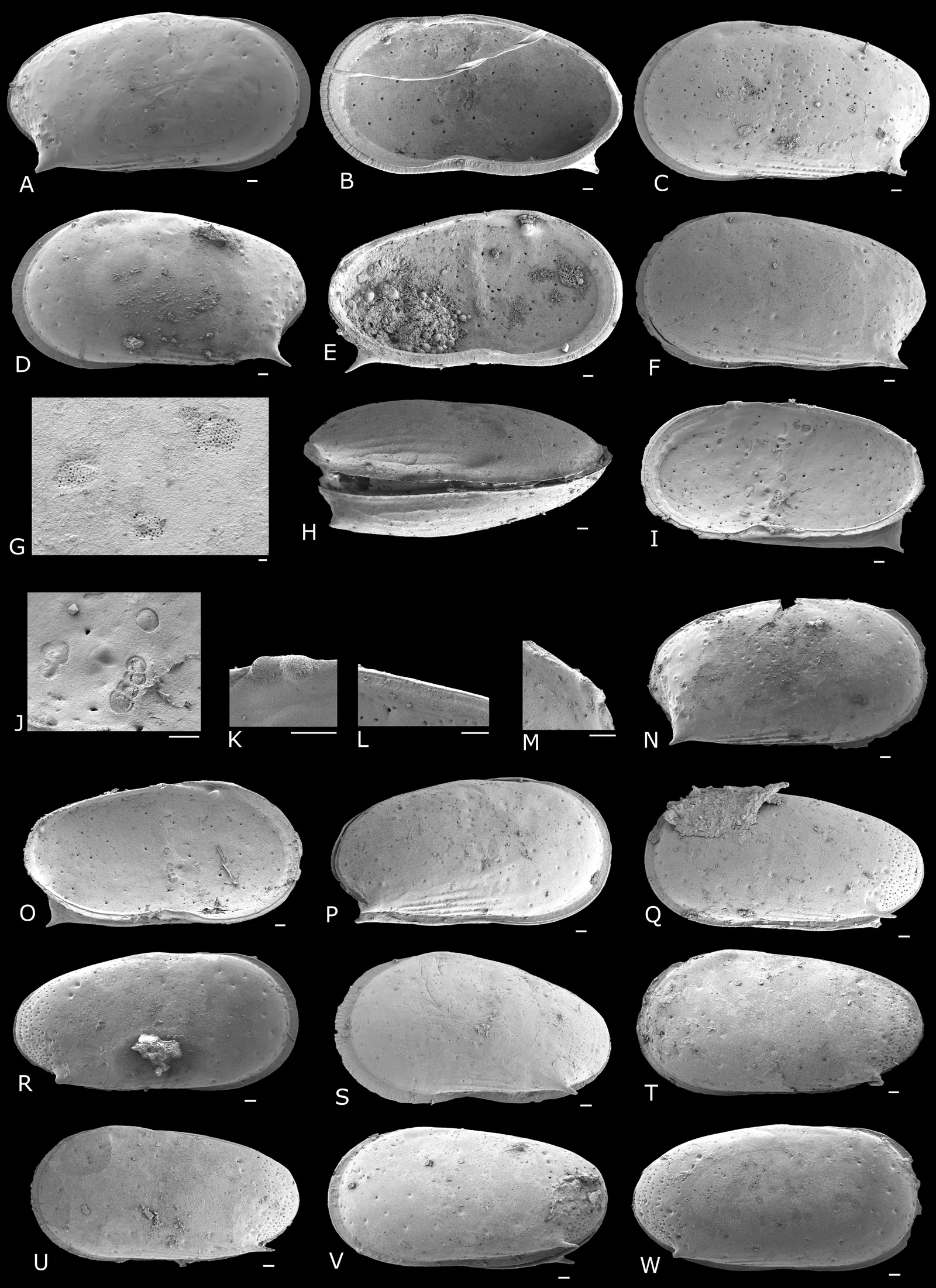
Scanning electron microscopic photographs of *Aothai kresnae* gen. nov. sp. nov. from the inner Gulf of Thailand. (A, B) Holotype, sample BK2-3, SUT-25KU-117. (A) RV in outer view. (B) inner view of the same specimen. (C) Paratype 1, sample BK2-3, SUT-25KU-114, LV in outer view. (D, E) Sample BK2-3, SUT-25KU-122. (D) LV in outer view. (E) Inner view of the same specimen. (F) G. Paratype 2, sample BK2-3, SUT-25KU-115. (F) Carapace in left outer view. (G) Detail of the surface of the same specimen showing sieved pores. (H) Sample BK2-3, SUT-25KU-120, carapace in ventral view. (I–N) Sample BK2-3, SUT-25KU-119. (I) RV in inner view. (J) Detail of the muscle scars. (K) Anterior element of the hinge. (L) Median finely crenulate socket. (M) Posterior element of the hinge sketch of hinge and muscle scars. (N) Outer view of the same specimen. (O) Sample BK2-3, SUT-25KU-121, LV in inner view. (P) Sample BK2-3, SUT-25KU-116, carapace in right lateral view. (Q, R) Sample BK2-3, SUT-25KU-130. (Q) Carapace in left lateral view. (R) Right lateral view of the same specimen. (S) Sample CP1-3, SUT-25KU-113, LV in outer view. (T) Sample BK2-3, SUT-25KU-131, LV in outer view. (U) Sample BK2-3, SUT-25KU-128, carapace in left lateral view. (V, W) Sample BK2-3, SUT-25KU-127. (V) carapace in left lateral view. (W) Right lateral view of the same specimen. All scale bars represent 20 µm, except R: 1 µm.

**Figure 7:**
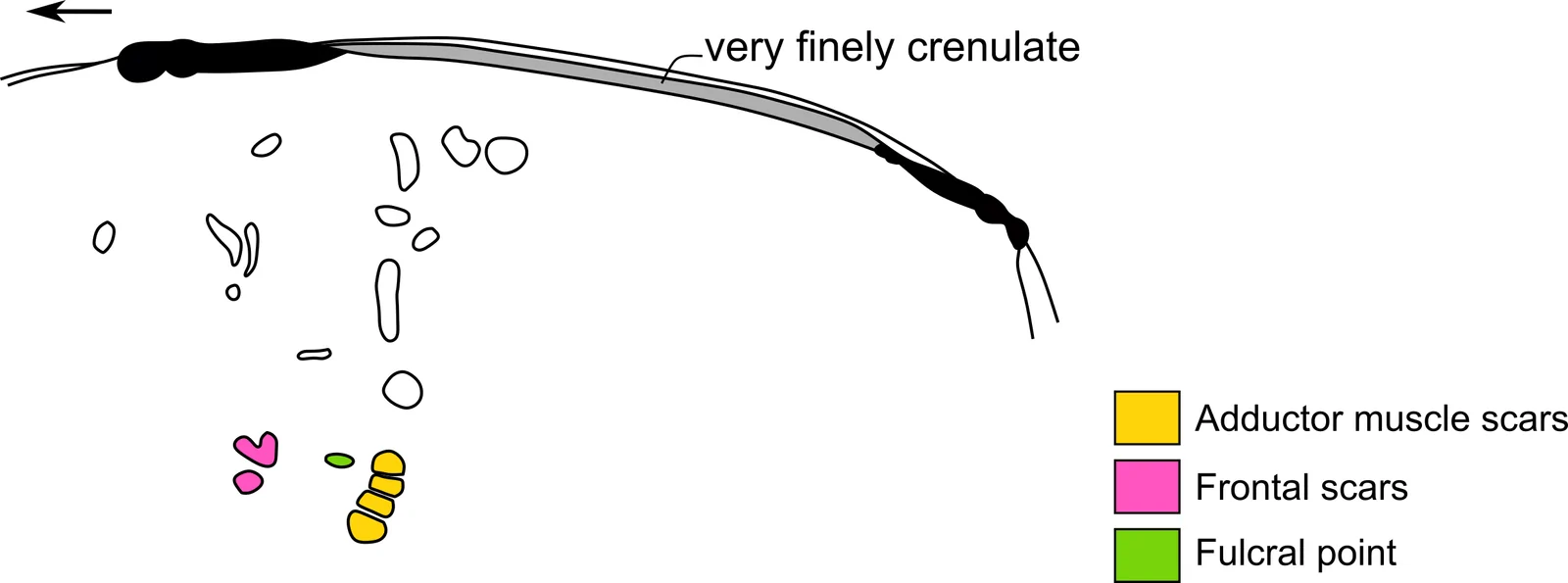
Interpretative sketch of hinge and muscle scars of *Aothai kresnae* gen. nov. sp. nov.

**Figure 8:**
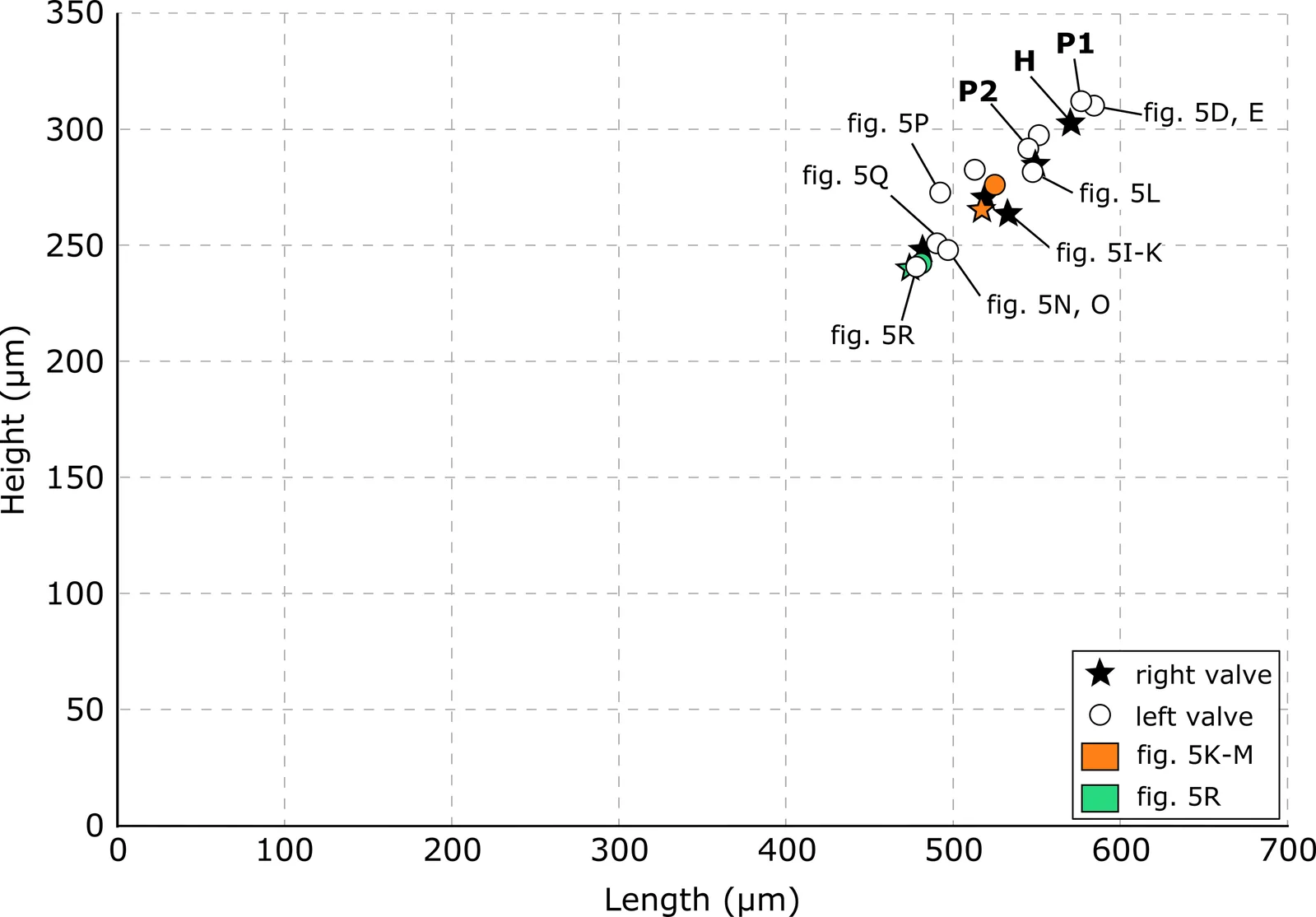
Height and length ratio of all specimens of *Aothai kresnae* gen. nov. sp. nov. from the present study. Isolated left valves are represented by white circles with black outlines; isolated right valves by black stars. For complete carapaces, left and right valves were measured separately and are represented in the same color. H, Holotype. P1, paratype 1. P2, paratype 2.

**Figure 9:**
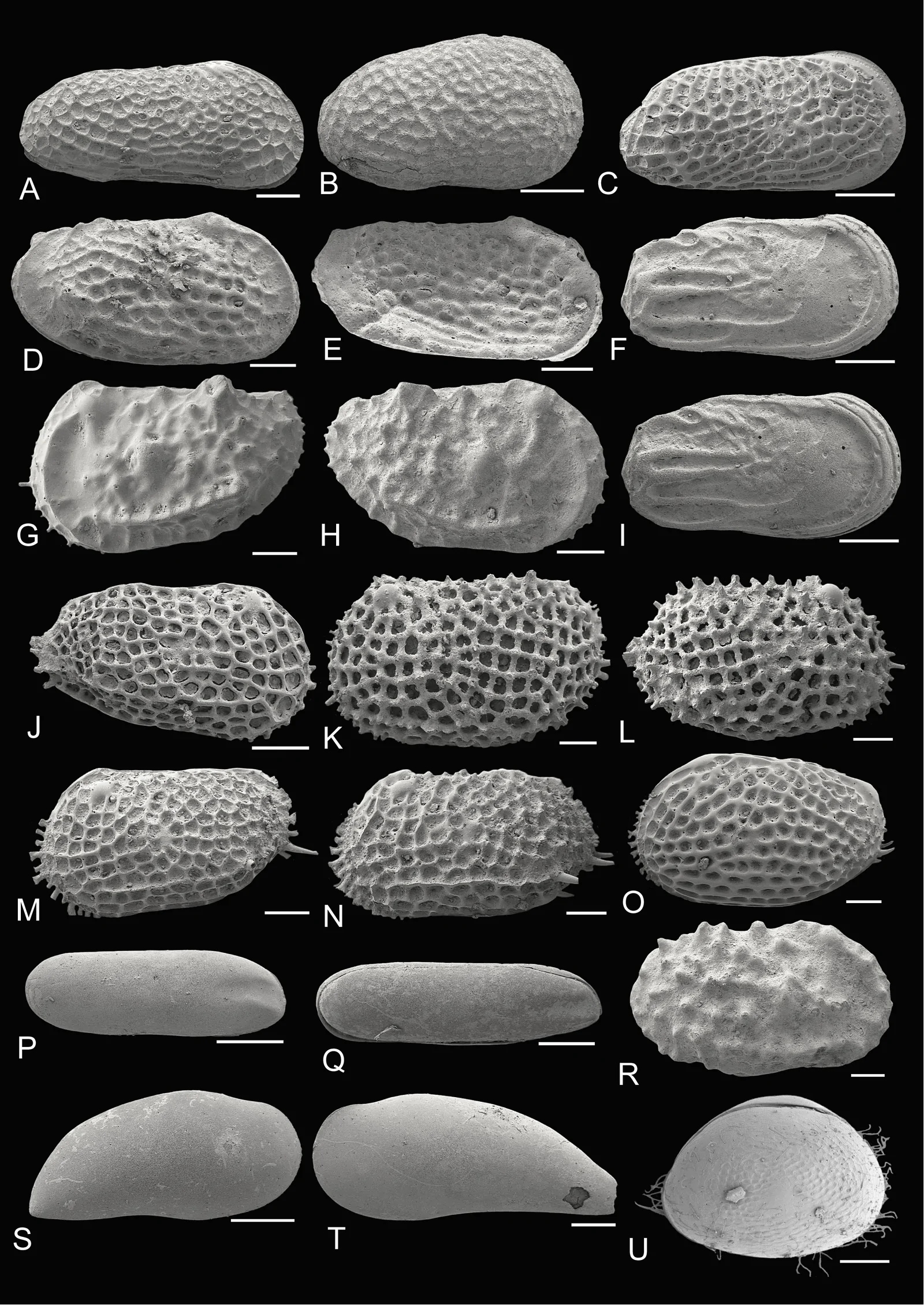
Scanning electron microscopic photographs of ostracods from the inner Gulf of Thailand. (A) *Hemicytheridea reticulata* Kingma, 1948, right valve, sample GT1, SUT-25KU-055. (B–C) *Hemicytheridea cancellata* (Brady, 1868). (B) Right valve, sample GT1, SUT-25KU-058. (C) Right valve, sample GT8, SUT-25KU-060; (D–E). *Neocytheretta murilineata* (Zhao & Whatley, 1989). (D) Right valve, sample GT3, SUT-25KU-062. (E) Right valve, sample GT6, SUT-25KU-064. (F, I) Atjehella sp. (F) right valve, sample GT1, SUT-25KU-081. (I) right valve, sample GT3, SUT-25KU-082. (G, H) Neocytheretta sp., (G) right valve, sample GT4, SUT-25KU-065. (H) Left lateral valve, sample GT4, SUT-25KU-066. (J) *Lankacythere multifora* Mostafawi, 1992, right valve, sample GT5, SUT-25KU-091. (K, L) *Lankacythere* sp. (K) Left valve, sample GT4, SUT-25KU-092. (L) Right valve, sample GT8, SUT-25KU-093. (M, N) *Pistocythereis subovata* Gou in Gou, Zheng & Huang, 1983, M, left valve, sample GT5, SUT-25KU-094. (N) Left valve, sample GT8, SUT-25KU-095. (O) *Venericythere papuensis* (Brady, 1880), left valve, sample GT1, SUT-25KU-097. (P, Q) *Copytus posterosulcus* Wang, 1985. (P) Left valve, sample GT6, SUT-25KU-099. (Q) Left view of a carapace, sample GT7, SUT-25KU-100. (R) *Stigmatocythere* sp., right valve, sample MK1, SUT-25KU-101. (S) *Phlyctenophora orientalis* (Brady, 1868), right valve, sample GT6, SUT-25KU-108. (T) Paracypris ? sp., right valve, sample GT5, SUT-25KU-111. (U) Gen indet., right view of a carapace, sample BK1, SUT-25KU-132. Scale bars = 0.1 mm.

## Results

### Taxonomy

**Table:** 

Subclass Podocopa Müller, 1894
Order Podocopida Müller, 1894
Suborder Cytherocopina Baird, 1850
Superfamily Cytheroidea Baird, 1850
Family Cytherideidae Baird, 1850
Subfamily Cytherideinae Sars, 1922–1928
Genus *Neocyprideis Apostolescu, 1956*
Type species. *Cyprideis* (*Neocyprideis*) *durocortoriensis*Apostolescu, 1956 by original designation.
*Neocyprideis agilis* (Guan et al., 1978)
Figs. 4I–4U

1978 *Cytheridea agilis* - GUAN, Paleontological Atlas of Central and South China, 4: 239-240, figs 12–14.

1985 *Cyprideis* sp. - CARBONEL, HOIBIAN and MOYES, Zone delta Yque de la Mahakam, pl. 3, figs 1–2.

2004 *Neocyprideis* sp. 1 - MONTENEGRO, PUGLIESE and SCIUTO, Mae Khlong river mouth, Thailand, pl. 2, fig. 1.

2005 *Neocyprideis agilis (Guan, 1978)* - WOUTERS, Java, Indonesia, pls.1-3.

2021 *Neocyprideis* sp. - FOREL, southwestern coast of Peninsular Thailand, Andaman Sea, pl. 1, fig. 11.

**Materials:** 797 isolated valves and 536 carapaces.

**Dimensions:** H = 0.44–0.55 mm; L = 0.71–0.85 mm; H/L = 0.62–0.64.

**Occurrence:** Yingli, Haikang County, China, Pliocene (Guan et al., 1978); Delta of the river Mahakam, Kalimantan, Indonesia (Carbonel, Hoibian & Moyes, 1985); Mae Khlong river mouth, Thailand (Montenegro, Pugliese & Sciuto, 2004); Pentai Kenjeran, Surabaya, Java, Indonesia (Wouters, 2005); coastal and marine areas of Hong Kong, China (Hong, 2016); Andaman Sea, Satun, Thailand (Forel, 2021); river mouths and the Gulf of Thailand (this study).

**Remarks:** *Neocyprideis agilis* is characterized by a strongly convex dorsal margin, with a distinct posterior cardinal angle, particularly in the left valve (LV). The anterior margin is broadly rounded, while the posterior margin is obliquely rounded. The ventral margin is straight in the right valve (RV) and slightly convex in the LV, with the LV being noticeably larger than the right one (Wouters, 2005). This species was first described from Pliocene materials in China (Guan et al., 1978) and has since been documented across the Indo-Pacific region, including in the northwestern Gulf of Thailand (Montenegro, Pugliese & Sciuto, 2004), Andaman Sea in Satun Province, Thailand (Forel, 2021) and as living specimens in Indonesia (Wouters, 2005).

The specimens of *Neocyprideis agilis* from the river-mouth sediments studied here display distinct morphologies. Juvenile specimens have a slender carapace with a straight dorsal border inclined steeply to the posterior border (Figs. 4S–4U) while adults have thicker and more rounded carapaces, which are similar to the specimens described from previous research (Figs. 4I–4R). The specimens of *N. agilis* in this study exhibit a wider range of carapace sizes compared to those living specimens described by Wouters (2005).

**Table:** 

Family Loxoconchidae Sars, 1922–1928
**Genus** ***Aothai*** ** Forel, Chitnarin, Weerachai, Ketwetsuriya gen. nov.**
**Type species.** ***Aothai kresnae*** ** Forel, Chitnarin, Weerachai, Ketwetsuriya gen. nov. sp. nov., by monotypy.**
**Zoobank link**.urn:lsid:zoobank.org:act:1834D6A5-CDAB-4B08-B926-627C30CE6AC0

**Diagnosis:** Carapace ovoid to subrectangular, with posterior margin narrowly rounded around or below mid-H, inflated in dorsal view. Free margin of both valves bordered by thin flange from antero-cardinal angle to lower part of posterior border. Each valve bears a short postero-ventral spine. Surface predominantly smooth with (1) faint reticulation in posterior area, (2) longitudinal ridges on postero-ventral surface with variably expressed intervening reticulation, (3) loose punctation. Surface scattered with sieved and unsieved normal pores. Eye tubercle absent. In inner view, adductor muscle scars in arcuate row of 4, frontal scar V-shaped. Fulcral point present. Hinge gongylodont, very delicate. Zone of concrescence narrow, more so posteriorly.

**Etymology**: From the Thai 
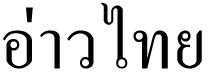
 (Ao Thai), for “Gulf of Thailand”, referring to the new genus being described from the Gulf of Thailand. Gender of the genus: feminine.

**Assigned species**: “Undetermined mature new species and genus” in Dewi (1997), here named *Aothai kresnae* gen. nov. sp. nov.

**Remarks**: The morphology of *Aothai* gen. nov. is very evocative of the genus *Hemikrithe* Bold van den, 1950 (type species: *Hemikrithe orientalis* Bold van den, 1950), whose familial attribution has been the subject of debate, as summarized by Malz (1982). *Hemikrithe* was originally placed in an uncertain family within the Cytheroidea (Bold van den, 1950; Moore, 1961; van Morkhoven, 1963) and later within Krithidae (Hartmann & Puri, 1974; Jain, 1978). It was finally related to the Trachyleberididae (Keij, 1979), though Dewi (1997) attributed it to Brachycytheridae Puri, 1954 and Arculacytherinae (Hartmann, 1981) without discussion. Malz (1982) made it clear that *Hemikrithe* is characterized by a crenulate hinge with an anti-slip tooth at the bottom of the anterior socket in the LV, a broad anterior duplicature, and a stop-knob mid-ventrally below the incurvature of the selvage of RV to receive the selvage of the overlapping LV (differing from the snap-knob fitting into the snap-pit of Leptocytheridae Hanai, 1957; *e.g.*, Gliozzi et al., 2005; Namiotko et al., 2012). Beyond superficial similarity with *Hemikrithe*, *Aothai* lacks the broad anterior duplicature and the mid-ventral stop-knob.

A LV (in inner view) and a RV identified as “Undetermined mature new species and genus” from Java Sea by Dewi (1993, Figs 238-240), are congeneric to *Aothai*. Dewi (1993, p. 152) described them as follows: “this new species and genus is characterized by a small posterior spine; the inner lamella has only a few normal pore canals; and a thin carapace (I. Yassini, 1992, personal communication). This species has a loxoconchid hinge, but its shape and carapace are similar to *Hemikrithe orientalis* (R.C. Whatley, 1993, personal communication)”. The loxoconchid hinge mentioned by Dewi (1997) corresponds to the gongylodont type of hinge observed here, which is typical of Loxoconchidae (*e.g.*, Cohen, Peterson & Maddocks, 2007). *Aothai* gen. nov. differs from all loxoconchid genera known to science by its postero-ventral spine on both valves, anterior flange and very delicate hinge.

**Table:** 

***Aothai kresnae*** ** Forel, Chitnarin, Weerachai, Ketwetsuriya gen. nov. sp. nov.**
** Zoobank link**. urn:lsid:zoobank.org:act:D722A8FE-CAA4-4A3E-8921-5FA8C61337E4
Figs. 6–8
*1993* Undetermined mature new species and genus; *Dewi*: p. 152, *Figs. 238–240*.

**Type material:** Holotype, a RV SUT25-KU-117, sample BK2-3 (Figs. 6A, 6B). Paratype 1, a LV SUT-25KU-114, sample BK2-3 (Fig. 6C). Paratype 2, a carapace SUT25-KU-115, sample BK2-3 (Figs. 6F, 6G).

**Additional material:** 5 RV, 5 LV, 6 carapaces.

**Type locality:** sample BK2, 13.422501N, 100.945837E, Bang Pakong River mouth, Gulf of Thailand.

**Etymology**: In reference to Dr. Kresna Tri Dewi (Indonesian Geological Survey), who observed the present species for the first time, as an undetermined new species and genus.

**Diagnosis**: As for genus.

**Description**: Carapace ovoid to subrectangular, tapered posteriorly in lateral view, wedge-shaped with maximum of width behind mid-length in ventral and dorsal views. LV overlaps RV along posterior and antero-ventral borders and at antero-dorsal concavity directly in front of mid-length (Fig. 6P). RV overlaps LV along postero-ventral border (Fig. 6V). Hmax located at anterior cardinal angle in LV, posterior to it in RV; maximum of length around mid-height in both valves. In both valves, anterior border symmetrically rounded, located around mid-height. Posterior border narrow, asymmetrically rounded, at mid-height or slightly below. In LV, dorsal margin long, straight and inclined posteriorly with an angle between 15° and 21° in the smallest specimens, with poorly marked yet visible cardinal angles. In RV, the dorsal margin is more convex with rather indistinct cardinal angles and a tenuous concavity in front of mid-length. Ventral margin long in both valves, with oral concavity around mid-length. On each valve:

– a thin and finely striated flange extends along free margin, from anterior cardinal angle to postero-ventral border, becoming very narrow within oral concavity;

– a postero-ventral spine slightly above ventral margin, short, thin and nearly horizontal in small specimens (Figs. 6Q–6W), thicker and triangular in large specimens (Figs. 6A–6O).

Surface of both valves predominantly smooth with:

– faint reticulation on posterior third of both valves;

– longitudinal ridges on postero-ventral surface with variably expressed intervening reticulation;

– loose punctation, scattered all over the surface. The smallest specimens display abundant punctae on the posterior surface behind postero-ventral spine (Figs. 6Q–6W).

Surface scattered with sieved pores (Fig. 6G), including a row of pores along the anterior margin, regularly spaced.

In the inner view, adductor muscle scars form an arcuate row of 4, with the upper and lower ones hemicircular and the median ones rectangular (Figs. 6I–6J, 7). Frontal scar V-shaped, accompanied by a subcircular scar located below it. Fulcral point in front of the uppermost adductor scar. Hinge gongylodont, thin, composed in RV of two bulbous teeth followed by a thin lamellar tooth, a long and very finely crenulate median socket, two small bulbous teeth, thin lamellar tooth and two larger bulbous teeth (Figs. 6I, 6K–6M, 7). Zone of concrescence narrow, larger anteriorly, absent in oral concavity and very thin posteriorly. Vestibulum reduced.

**Dimensions:** (Fig. 8). Holotype: H = 537 µm, L = 303 µm. Paratype 1: H = 577 µm, L = 312 µm. Paratype 2: H = 584 µm, L = 310 µm.

**Remarks**: *Aothai kresnae* gen. nov. sp. nov. is so far the only known species of the genus. The external surface of this species displays intraspecific variations related to its ontogenetic development. Juveniles, as indicated by their small size, lower and very thin valves, and weakly developed internal features (hinge and muscle scars), display fine punctation on the posterior end behind the postero-ventral spine, with punctae roughly arranged parallel to the posterior border (Figs. 6Q–6W). On submature and adult specimens, punctae are absent and replaced by faint, loose reticulation, the muri displaying an arrangement similar to that of the punctae in juveniles (Figs. 6A–6P). Juveniles also exhibit weaker but distinct longitudinal ridges on the postero-ventral surface.

**Occurrences:** Stations 38, 55, 53 and core 14 (2.5–5 cm), Java Sea (Dewi, 1993). River mouth of Mae Khlong (MK1, MK2), Chao Phraya (CP1), Bang Pakong (BK2) and sediment surface of sample GT3, Gulf of Thailand (Table 4 and Tables S1, S2; Fig. 1; this work).

### Physical characteristics of the sampling sites

The sampling stations were classified into river-mouth and offshore settings, with the former directly influenced by freshwater discharge and the latter located farther from the coastline. Water depths at the river-mouth stations were shallow, ranging from 0 to -8 m, whereas the offshore stations ranged from −7 to −23 m. Sediments at all sites were predominantly fine-grained, comprising silt and clay, whereas fine-grained sands were found in MK1, GT1, GT2, GT3, GT4, and GT6 (Table 1). Samples MK1-MK3 consist of fine-grain sand, silt, and clay; TC1, TC2, TC3 are silt and clay; CP1-CP3, BK1-BK3 are finer, mainly clay. Mollusk shells are found in river mouth sediments, especially in BK1-BK3, which contain many 0.5–1.5 cm-sized bivalve shells. In the Gulf, surface samples GT2-GT4 and GT6 and 10-cm-deep samples GT1, GT2, and GT6 consist of fine-grained sand, silt, and clay. Other samples consist of silt and clay. Some samples from the offshore Gulf are thus slightly coarser than the river-mouth samples, suggesting a potential influence of tidal action and sediment transport dynamics in the offshore environment.

Seawater environmental variables at the sampling stations were measured to assess the influence of marine conditions on sediment composition. Figure 3 illustrates the temperature variations (°C), dissolved oxygen (mg/L), pH, and salinity (PSU) levels at the various sampling sites. Seawater temperature in the Gulf of Thailand (GT1-GT8) fluctuated between the two sampling periods. However, it ranges from 29.8 to 33.3 °C, and the surface temperature is generally higher than at deeper levels (Fig. 3A). Dissolved oxygen ranges from 4.16 to 8.66 mg/L; levels in December are higher than in May except for the deep level at GT1 (Fig. 3B). The pH levels are normal, ranging from 7.79 to 8.61 (Fig. 3C). The salinity varies from 28.2 to 33.4 PSU, representing normal marine conditions (Fig. 3D).

All parameters measured at the surface and deep levels (GT samples) were generally similar, but differences can be observed between sampling periods. Generally, the dissolved oxygen and salinity levels in December were higher than in May, but the pH levels were higher in May. However, the temperatures measured in May oscillated due to the NE monsoon during the sampling. Samples located near the shore, especially for GT1, had variations in salinity, pH, temperature compared to those of samples GT5-GT8, which were farther offshore.

The temperature measured at the river mouth stations fluctuated between 27.2 and 35.1 °C (Fig. 3E), recorded in December and May, respectively. Dissolved oxygen levels ranged from 4.40 to 7.30 mg/L, with the lowest values recorded at BK stations (Fig. 3F). pH ranged from 7.38 to 10.73, with the maximum value recorded at CP3 in May (Fig. 3G). The salinity ranged from 9.18 to 30.92 PSU. The salinity measured in May, which was lower than 26 PSU, was influenced by precipitation and runoff at the beginning of the rainy season. The 9.18 and 9.56 PSU were recorded at MK3 and CP2 stations (Fig. 3H).

Variations in environmental parameters among stations (Tables 1–3) are generally subtle; however, differences in depth and salinity are distinguishable. Salinity at river-mouth stations is typically below 30 PSU, with the lowest value (9.18 PSU) recorded at station MK3 in May 2023. In contrast, offshore stations generally exhibit salinity values above 30 PSU, except for December measurements at stations GT1 and GT2 (28.2 and 29.4 PSU, respectively).

Environmental conditions at the Mae Klong stations show relatively narrow ranges: temperatures varied from 29.7–32.3 °C in May and 27.2–28.0 °C in December; dissolved oxygen ranged from 5.6–6.1 mg/L in May and 5.8–7.3 mg/L in December; and pH values ranged between 7.69 and 8.05. However, salinity ranged from 9.18 to 28.32 PSU, with the minimum value recorded at MK3 in May 2023 (Table 2; Fig. 3).

### Ostracod diversity

Forty species are recovered from the sediment samples, and a new genus and species are proposed (Figs. 4–6, 9). The species recovered in this study have previously been reported from the Indo-Pacific region, including China, Hong Kong, Vietnam, Indonesia, Malaysia, and Thailand (Phetchaburi, Krabi, Satun). The occurrences at the river mouth and the Gulf stations are shown in Table 4, Tables S1 and S2. The relative abundance and occurrences of the most abundant species found in this study are shown in Fig. 10.

Twenty-three species of ostracods were found in the river mouth sediments. Among these river mouths, ostracods were most abundant in sediments from the Mae Klong River. A total of 341 specimens were recorded in May 2023 and 632 in December 2023 (973 specimens in total). Twenty species were identified. The assemblage is strongly dominated by *Neocyprideis agilis* (700 specimens), followed by *Tanella gracilis* (57 specimens) and *Neocytheretta* sp. (49 specimens), with additional occurrences of *Keijella gonia*, *K. multisulcus*, and *Stigmatocythere bona* (Table S2). The sampling sites are characterized by very shallow water depths (0 to −3 m) with silts, clays, and fine-grained sands (only at MK1).

Sediments from the Bang Pakong River yielded 171 ostracod specimens in May and 190 specimens in December (361 specimens in total). The assemblage is again dominated by *N. agilis* (281 specimens), followed by *Aothai kresnae* (25 specimens). In contrast, sediments from the Chao Phraya and Tha Chin rivers yielded comparatively few specimens (31 specimens and 38 specimens, respectively). Notably, sediments at the Mae Klong and Tha Chin river mouths are coarser than those from the Chao Phraya and Bang Pakong systems. Several taxa, including *Paracathaycythere cf. costaereticulata*, *Neomonoceratina mediterranea malayensis*, *Carinocythereis* sp., *Stigmatocythere* sp., and *N. agilis*, are restricted to river-mouth environments.

Thirty-three species were recovered from the offshore Gulf sediments, yielding higher ostracod diversity (Table S1), and the number of specimens recovered from surface sediments and at 10 cm depth were generally similar. The assemblage is dominated by *Keijella multisulcus*, followed by *Paranesidea* sp. and *Hemicytheridea cancellata*. Several taxa—such as *Cytherella incohata*, *Paranesidea* sp., *Javanella kendengensis*, *Neocytheretta murilineata*, *Atjehella* sp., *Lankacythere* spp., *Pistocythereis subovata*, *Venericythere papuensis*, *Copytus posterosulcus*, *Phlyctenophora orientalis*, and *Paracypris?* sp.—are absent from river-mouth settings.

**Figure 10:**
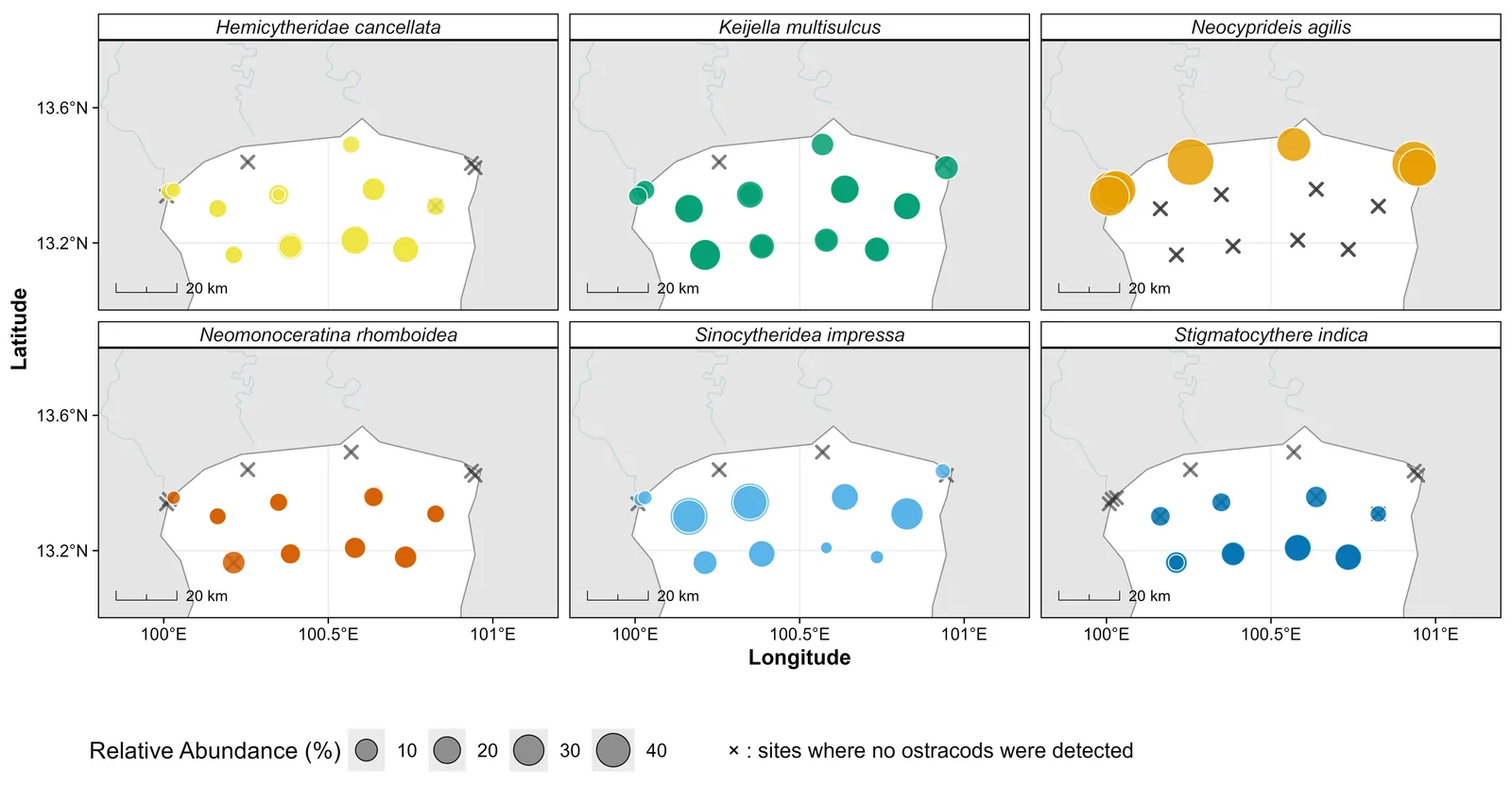
Spatial distribution and relative abundance of the six most abundant species in the Gulf of Thailand and adjacent river mouths.

## Discussion

### Ostracods and environmental parameters

The ostracod assemblage observed in this study is comparable to those reported by Montenegro, Pugliese & Sciuto (2004) and Pugliese et al. (2006) from shallow marine environments near the Mae Klong River mouth, where depth and substrate were defined as primary controls on species distribution. In their study, ostracods were absent in sediments interpreted as tidal channels, from very shallow waters (1–4 m depth) with sandy sediments, low salinity (20–22 ppt), low dissolved oxygen (3–3.2 mg/L), and low pH (5.7–5.8). On the contrary, ostracods were generally found off the tidal flats, in sediments at depths of 1–18 m, silt to silty sand (rare in sandy) sediments, and with salinity ranging from 18–32 ppt. They reported that species such as *Keijella gonia*, *K. multisulcus*, *Neomonoceratina iniqua*, *Sinocytheridea impressa (Brady, 1869)*, *Stigmatocythere bona, Hemikrithe orientalis Bold, 1950, Neosinocythere elongata (Hu, 1976) Zhao (Yi-Chun) & Whatley, 1993, Cytherella hemipuncta Swanson, 1969* were recovered from all depths. This is confirmed by our study, which shows that *K. gonia, K. multisulcus*, *S. impressa*, *S. bona, and H. orientalis* are distributed from the river mouth to offshore environments. Pugliese et al. (2006) reported that *Neocytheretta murilineata, Hemicytheridea cancellata (Brady, 1868), Tanella gracilis Kingma, 1948*, *Phlyctenophora orientalis*, and *Neocyprideis* sp.1 were limited to shallow-water environments (1–6 m); however, these species, except *N.* sp.1, are found in our offshore sediments. In our recent study, we recovered many specimens of *Neocyprideis agilis*, and we certainly included *N.* sp. 1 *sensu* Montenegro, Pugliese & Sciuto (2004). The high abundance of *N. agilis*, particularly the presence of well-preserved complete carapaces of adults and juveniles, further supports its preference for shallow to very shallow habitats (Fig. 10). Our results support that *Stigmatocythere indica (Jain, 1977)* and *Copytus posterosulcus* prefer the depth below 5 m, as they were recovered from our offshore sediments.

### Taxonomic richness and biogeographic significance

The recovery of 40 ostracod species demonstrates substantial taxonomic richness in the inner Gulf of Thailand and associated river-mouth environments. Importantly, all identified species have been previously documented from Indo-Pacific regions, including China, Hong Kong, Vietnam, Indonesia, Malaysia, and Thailand, confirming a broad faunal continuity across this marine province (*e.g.*, Carbonel, Hoibian & Moyes, 1985; Montenegro, Pugliese & Sciuto, 2004; Wouters, 2005; Hong, 2016; Forel, 2021).

As this study represents the first comprehensive documentation of ostracods from the inner Gulf of Thailand—apart from the work of Montenegro, Pugliese & Sciuto (2004)—we propose that the dispersal of ostracods within this region may be influenced by seasonal surface circulation patterns. Previous studies (*e.g.*, Sojisuporn, Morimoto & Yanagi, 2010) have shown that surface currents in the Gulf are strongly controlled by monsoonal forcing: during the southwest (SW) monsoon, surface waters predominantly flow northeastward, whereas during the northeast (NE) monsoon, circulation reverses direction (Fig. 2). Such seasonal reversals may provide potential pathways for the transport of marine organisms, including benthic ostracods, across the region. In particular, the SW monsoon-driven currents could facilitate northward dispersal from lower latitudes, potentially connecting faunal assemblages from Malaysia and Indonesia to the Gulf of Thailand. This mechanism may help explain the occurrence of *Aothai kresnae*, previously reported from Java, Indonesia, within our study area. Conversely, during the NE monsoon, surface currents flow from the open South China Sea into the Gulf of Thailand, with subsequent circulation directed toward the inner Gulf. This pattern may enable the introduction and redistribution of ostracod species originating from the South China Sea into more restricted inner Gulf environments.

However, the observed distribution patterns in this study indicate that several species are confined either to river-mouth settings or to offshore environments. This ecological partitioning suggests that, although large-scale current systems may contribute to regional dispersal, local environmental controls such as salinity gradients, substrate type, and water depth likely play a critical role in limiting species establishment and distribution. Consequently, the relative importance of physical transport *versus* ecological filtering in shaping ostracod assemblages in the inner Gulf of Thailand remains uncertain and warrants further investigation.

## Conclusions

This study provides the first comprehensive assessment of modern ostracod assemblages inhabiting the inner Gulf of Thailand and its associated river mouths, revealing both taxonomic richness and spatial ecological differentiation. A total of 40 species were identified, and one new genus and species were described. The dominance of *Neocyprideis agilis* at the river mouths, where all ontogenetic stages were recovered, underscores its ecological importance as a euryhaline species that tolerates variable salinity and fine-grained sedimentary environments.

Despite physical and chemical variability between the May and December 2023 sampling periods, no clear seasonal changes in ostracod community composition were observed. The fluctuations in salinity and temperature in the Gulf of Thailand may not influence the ostracod community in different seasons.

The findings suggest that benthic ostracod communities exhibit clear spatial differentiation associated with local sedimentary and hydrochemical conditions. The discovery of a new species demonstrates that taxonomic studies are still necessary on tropical shelves.

##  Supplemental Information

10.7717/peerj.21817/supp-1 Supplemental Information 1 Distribution and number of ostracods in the inner Gulf of Thailand samples

10.7717/peerj.21817/supp-2 Supplemental Information 2 Distribution and number of the ostracods in the river mouth samples

## Additional Information and Declarations

### Field Study Permissions

The following information was supplied relating to field study approvals (*i.e.*, approving body and any reference numbers): Fieldwork and sample collection were conducted by researchers and students from Kasetsart University as part of an academic research project. The sampling sites were public coastal and estuarine areas where no special permit was required under Thai regulations.

### Data Availability

The following information was supplied regarding data availability: The raw measurements are available in the Supplemental Files.

### New Species Registration

The following information was supplied regarding the registration of a newly described species: Genus name: urn:lsid:zoobank.org:act:1834D6A5-CDAB-4B08-B926-627C30CE6AC0 Species name: urn:lsid:zoobank.org:act:D722A8FE-CAA4-4A3E-8921-5FA8C61337E4 Publication LSID: urn:lsid:zoobank.org:pub:083A1912-6302-4BCA-A412-D6EB0753FDDB
